# Supplementary material for: Comparing methods for plasma HDV RNA quantification in bulevirtide-treated and untreated patients with HDV
Source: JHEP Rep. 2024 Dec 11;7(3):101299. doi: 10.1016/j.jhepr.2024.101299 (PMC11883403; doi:10.1016/j.jhepr.2024.101299)
Supplement: Multimedia component 1 [file mmc1.pdf]

Comparing methods for plasma HDV RNA quantification in bulevirtide-treated and untreated patients with HDV

Maria Paola Anolli, Sara Uceda Renteria, Elisabetta Degasperi, Floriana Facchetti, Dana Sambarino, Marta Borghi, Riccardo Perbellini, Roberta Soffredini, Sara Monico, Annapaola Callegaro, Pietro Lampertico

Table S1……………………………………………………………………………...…..2

Table S2a……………………………………………………………………………..4

Table S2b……………………………………………………………………………..4

Table S1. Main demographic, biochemical and virological features of the 130 patients included in the study

Variables	Robogene *vs.* EurobioPlex Patients n=127	Robogene *vs.* AltoStar Patients n=44
Age, years	52 (23-77)	51 (29-77)
Males	68 (54%)	25 (57%)
BMI, kg/m^2	24 (17-41)	24 (19-41)
European origin	116 (91%)	42 (96%)
HDV Genotype 1*	108 (96%)*	44 (100%)*
Compensated cirrhosis†	79 (62%)	44 (100%)
Ongoing NUC treatment	95 (75%)	44 (100%)
Previous PegIFNα treatment▪	63 (50%)	23 (52%)
AST, U/L	63 (16-374)	46 (18-592)
ALT, U/L	76 (6-743)	43 (12-684)
PLT, $10^3/mm^3$	146 (29-344)	80 (37-220)
LSM kPa°	11.1 (4.0-62.1)	16.5 (7.2-62.1)
Esophageal varices§	30 (41%)§	24 (57%)
qHBsAg, Log IU/mL	3.8 (0.3-4.6)	3.7 (0.3-4.4)
HbeAg negative	111 (87%)	42 (95%)
HBV DNA undetectable✩	93 (73%)	42 (95%)

*Genotype available in 112 (89%) and 44 (100%) patients

† Child Pugh Score > A in 11 (14%) patients

° Fibroscan® available in 102 (80%) and 44 (100%) patients

§EGD available in 73 (58%) and 44 (100%) patients

✡ <LLOQ, i.e. <10 UI/mL

Results are reported as number (percentage) or median (range).

BMI, body mass index; HDV, Hepatitis D Virus; NUC, nucleos(t)ide analogue; PegIFNα, Pegylated Interferon alpha; AST, aspartate aminotransferase; ALT, alanine aminotransferase; PLT, platelets; LSM, liver stiffness measurement; qHBsAg, Hepatitis B surface Antigen; HbeAg, Hepatitis B e-Antigen

Table S2a. HDV RNA levels quantified by both Robogene 2.0 and EurobioPlex in 232 samples

	EurobioPlex		
**Robogene 2.0**	**TND (n=39)**	**<LOD/LOQ (n=20)**	**DETECTED (n=166)**
TND (n=40)	29 (73%)	5 (13%)	6 (15%)[1]
<LOD (n=14)	11 (79%)	2 (14%)	1 (7%)[2]
Detected (n=178)	6 (3%)[3]	13 (7%)[4]	159 (89%)

[1] Median HDV RNA 2.29 (2.02-3.38) Log IU/mL; [2] HDV RNA 3.30 Log IU/mL; [3] Median HDV RNA 1.80 (1.32-2.49) Log IU/mL; [4] Median HDV RNA 2.25 (0.85-2.59) Log IU/ml

Table S2b. HDV RNA levels quantified by both Robogene 2.0 and AltoStar in 246 samples

	AltoStar		
**Robogene 2.0**	**TND (n=1)**	**<LOD (n=8)**	**DETECTED (n=237)**
TND (n=9)	1 (11%)	4 (44%)	4 (44%)[1]
<LOD (n=17)	0 (0%)	3 (18%)	14 (82%)[2]
Detected (n=220)	0 (0%)	1 (0.5%)[3]	219 (99.5%)

[1]Median HDV RNA 1.50, [2]median HDV RNA levels of 1.34 (1.00-2.03) Log IU/mL, [3]HDV RNA 0.85 IU/mL
