# Supplementary material for: Comparing methods for plasma HDV RNA quantification in bulevirtide-treated and untreated patients with HDV
Source: JHEP Rep. 2024 Dec 11;7(3):101299. doi: 10.1016/j.jhepr.2024.101299 (PMC11883403; doi:10.1016/j.jhepr.2024.101299)
Supplement: Multimedia component 4 [file mmc4.pdf]

Comparing methods for plasma HDV RNA quantification in bulevirtide-treated and untreated patients with HDV

Maria Paola Anolli[1,2], Sara Uceda Renteria[3], Elisabetta Degasperi[1,2], Floriana Facchetti[1], Dana Sambarino[1], Marta Borghi[1], Riccardo Perbellini[1], Roberta Soffredini[1], Sara Monico[1], Annapaola Callegaro[3], Pietro Lampertico[1,2,4,*]

JHEP Reports **2025**. vol. 7 | 1–9

Check for updates

Background & Aims: Accurate HDV RNA quantification is crucial for diagnosis and management of chronic hepatitis delta (CHD), yet a significant variability between assays exists. We compared three methods to quantify HDV RNA levels in untreated and bulevirtide (BLV)-treated patients with CHD.

Methods: Frozen plasma from untreated and BLV-treated patients with CHD were tested in a single-center retrospective study using three different assays: Robogene 2.0 HDV RNA Quantification Kit 2.0 (Roboscreen GmbH; limit of detection [LOD] 6 IU/ml on 7500 Fast Real-Time PCR System [Applied Biosystem]), EurobioPlex HDV PCR quantitative kit (Eurobio Scientific; LOD 100 IU/m) on CFX96™ real-time PCR detection system [Bio-Rad]), and AltoStar HDV RT-PCR RUO Kit 1.5 (Altona Diagnostics; estimated LOD <10 IU/ml) on the AltoStar®AM16.

Results: Overall, 431 plasma samples from 130 patients with CHD (69 untreated and 61 BLV-treated) were studied. Compared with Robogene 2.0, EurobioPlex reported higher HDV RNA levels (3.78 [0.70–7.99] *vs*. 4.69 [2.00–8.19] log IU/ml, p <0.0001), with viremia higher than >0.5 log in 160 (69%). Likewise, HDV RNA levels were higher with AltoStar than with Robogene 2.0 (3.32 [0.70–7.37] *vs*. 3.91 [0.19–7.54] log IU/ml, p <0.0001), with AltoStar reporting HDV RNA levels >0.5 log in 127 (52%). Although virological response rates (≥2 log decline *vs.* baseline) at Weeks 24 (Robogene 2.0 *vs.* EurobioPlex and AltoStar) and 48 (Robogene 2.0 *vs.* AltoStar) were similar across assays, rates of HDV RNA undetectability significantly differed between the three assays at Weeks 24 and 72 (p = 0.003 and p = 0.02, respectively).

Conclusions: HDV RNA levels quantified by EurobioPlex and AltoStar were 1 and 0.5 logs higher than those quantified by Robogene 2.0, respectively. HDV RNA undetectability rates during BLV treatment were assay-dependent.

© 2024 The Authors. Published by Elsevier B.V. on behalf of European Association for the Study of the Liver (EASL). This is an open access article under the CC BY license (http://creativecommons.org/licenses/by/4.0/).

Introduction

Chronic hepatitis delta (CHD) is the most impairing form of chronic viral hepatitis.[1–5] The initial stage in the diagnosis of CHD involves identifying total antibodies against the hepatitis delta antigen (anti-HD). However, to differentiate between CHD and past infection, it is necessary to perform a test for HDV RNA in serum or plasma. The aim of treating any form of chronic viral hepatitis is to prevent the development of liver-related events (*i.e.* hepatocellular carcinoma and hepatic decompensation) and, in the end, liver-related death. To assess treatments' efficacy, surrogate endpoints have been developed for chronic hepatitis B and C[6,7]; however, CHD lags behind. Some studies have addressed HDV viremia as a surrogate marker of hepatic disease progression, specifically cirrhosis development and hepatocellular carcinoma occurrence.[8–10] Based on these findings, the joint EASL–AASLD conference for HDV as well as the FDA identified HDV RNA levels as a surrogate endpoint for evaluating HDV treatments' efficacy.[11] Consequently, in clinical trials evaluating new treatment approaches for HDV, a 'virological response' has been defined as at least a 2 log HDV RNA reduction or HDV RNA undetectability, compared with baseline.[12]

Despite the paramount importance of HDV RNA monitoring in clinical practice and trials, broad variability exists in terms of HDV RNA quantification assays and nucleic acid extraction methods.[13–15] The 1st World Health Organization (WHO) International Standard for HDV RNA quantification was implemented in 2012, but it is valid only for HDV genotype 1.[16] The lack of a standardized and reliable quantification assay has led many researchers to develop in-house PCR tests for HDV RNA quantification.[17] In 2020, the EMA conditionally approved bulevirtide (BLV), a first-in-class entry inhibitor of HDV/HBV, at a dose of 2 mg daily, for the treatment of adult patients with compensated CHD.[18]

Recently, a new version of the EurobioPlex quantification assay has been developed, and its performances have been compared with those of the former version, with high levels of

* Corresponding author. Address: Division of Gastroenterology and Hepatology; Foundation IRCCS Ca' Granda Ospedale Maggiore Policlinico; Via F. Sforza 35, 20122 Milan, Italy. Tel.: +39-0255035432.
E-mail address: pietro.lampertico@unimi.it (P. Lampertico).
https://doi.org/10.1016/j.jhepr.2024.101299

reproducibility and repeatability. However, the new assay read approximately 0.88 log IU/ml less than the former. According to the WHO standards, the new assay gave results very close to their theoretical values.[19]

The aim of this study was to compare the performances of three widely used HDV RNA quantification assays (Robogene 2.0, EurobioPlex, and AltoStar) in untreated and BLV-treated patients with CHD.

Patients and methods

Patient population

This is a single-center retrospective study performed at the outpatient Liver Clinic of the Gastroenterology and Hepatology Division of the Foundation IRCCS Ca' Granda Ospedale Maggiore Policlinico in Milan, Italy. Frozen plasma samples from consecutive untreated and BLV-treated patients with CHD were analyzed. Patients provided informed consent to make their clinical records available and retrospectively assess HDV RNA on stored plasma samples. This study was approved by the local institutional review board and conformed to the 1975 Declaration of Helsinki.

HDV RNA quantification

HDV RNA levels were quantified using the Robogene HDV RNA kit 2.0 (Roboscreen GmbH, Leipzig, Germany; lower limit of detection [LOD] 6 IU/ml, linear range 5 to 10^8 IU/ml) on 7500 Fast Real-Time PCR System (Applied Biosystem, Schwerte, Germany), EurobioPlex HDV qRT-PCR EBX-004 (Eurobio Scientific, Les Ulis, France, LOD/lower limit of quantification (LOQ) 100 IU/ml, linear range 10^2–10^8 IU/ml) on the CFX96™ real-time PCR detection system (Bio-Rad, Hercules, California, USA), and the AltoStar HDV RT-PCR Kit 1.5 (Altona Diagnostics, Hamburg, Germany; estimated LOD <10 IU/ml, estimated LOQ 20 IU/ml). The linear range for the AltoStar HDV RT-PCR Kit 1.5 is not specified in the insert package using purification kit PK 15-45 on the AltoStar®AM16. Robogene 2.0 and EurobioPlex are CE-IVD real-time PCR kits for quantification of HDV RNA, applying the 1st WHO International Standard for HDV RNA (PEI code 7657/12). AltoStar is still a "research use only" (RUO) test. Robogene 2.0 is the one currently used in clinical trials for HDV antiviral treatments.[20] Nucleic acids were extracted according to the manufacturer's instructions, with a manual extraction method for Robogene 2.0 and EurobioPlex assays: INSTANT Virus RNA/DNA kit (Roboscreen GmbH) for Robogene 2.0 and NucleoSpin® Dx Virus Kit (Macherey-Nagel GmbH, Düren, Germany) for EurobioPlex. Conversely, the extraction method recommended for AltoStar is automated extraction using the AltoStar® Purification Kit 1.5 (Altona Diagnostics GmbH). The extraction input volume was 400 µl *vs.* 150 µl *vs.* 500 µl of plasma for Robogene 2.0 *vs.* EurobioPlex *vs.* AltoStar, respectively. The elution volume was 60 µl *vs.* 50 µl *vs.* 80 µl of Dnase/Rnase free water, respectively.

The LOD was defined as the lowest concentration at which 95% of all replicates test positive, LOQ was defined as the level below which HDV RNA cannot be reliably quantified, and thus, it is not possible to determine if HDV RNA levels are greater than, equal to, or below the LOD.[15]

HDV genotype was assessed using Sanger sequencing and analyses of the hepatitis delta antigen region, as previously described.[21]

All samples were stored at -80 °C. Samples were collected between January 2021 and July 2023, when the data lock was set. Samples were tested over a 3-month period after the data lock. The 1st WHO International Standard for Hepatitis D Virus RNA for Nucleic Acid Amplification Techniques (NAT)-Based Assays (PEI code 7657/12) provided by the Paul-Ehrlich-Institut (Langen, Germany) was reconstituted in 0.5 ml of sterile nuclease-free water before use according to manufacturer indications. Two panels of six samples were tested in replicate and analyzed with Robogene 2.0 and EurobioPlex assays. Sample 1 (S1) was the WHO International Standard for HDV RNA, and samples 2–5 (S2–S5) were obtained by performing a series of 1 $\log_{10}$ dilution steps starting S1.

A panel of five samples was tested in duplicate and analyzed using the AltoStar HDV RT-PCR Kit 1.5 assay. Samples were obtained by diluting the WHO International Standard for HDV RNA, with viral loads of 5×10^4, 5×10^3, 5×10^2, 5×10^1, 1×10^1, and 0.5×10^1.

HBV DNA was quantified using the Cobas® HBV Test on the Cobas® 4800 System (Roche Diagnostics, Mannheim, Germany; LOQ 10 IU/ml). Serum HBsAg was quantified using the Elecsys HBsAg II quantitative assay on the Cobas® e801 Analyzer (Roche Diagnostics GmbH, Mannheim, Germany; LOQ 0.05 IU/ml).

Study endpoint

The primary endpoint of the study was to compare plasma HDV RNA levels quantified by three different quantification assays in untreated and BLV-treated patients with CHD. As an exploratory endpoint, we compared HDV RNA levels measured by the three assays with the WHO HDV RNA International Standard.

Statistical analysis

Categorical variables are presented as frequencies (percentages) and continuous variables as median (range). Categorical variables

Table 1. Main demographic, biochemical, and virological features of the 130 patients included in the study.

Variables	Patients (N = 130)
Age (years)	52 (23–77)
Males	69 (53)
BMI (kg/m²)	24 (17–41)
European origin	118 (91)
HDV genotype 1*	111 (97)
Compensated cirrhosis†	82 (63)
Ongoing NUC treatment	98 (75)
Previous PegIFNα treatment	63 (49)
AST (U/L)	52 (16–592)
ALT (U/L)	70 (6–743)
PLT (10^3/mm³)	149 (29–344)
LSM (kPa)‡	14.1 (4.0-62.1)
Esophageal varices§	32 (43)
qHBsAg (log IU/ml)	3.8 (0.3–4.6)
HBeAg-negative	114 (88)
HBV DNA undetectable¶	95 (73)

ALT, alanine aminotransferase; AST, aspartate aminotransferase; LOQ, lower limit of quantification; LSM, liver stiffness measurement; NUC, nucleos(t)ide analog; PegIFNα, pegylated interferon alpha; PLT, platelets; qHBsAg, quantitative HBsAg.
Results are reported as n (%) or median (range).
*Genotype available in 115 (88%).
†Child-Pugh score >A in 11 (13%) patients.
‡Fibroscan® available in 105 (81%) patients.
§Esophagogastroduodenoscopy available in 75 (58%) patients.
¶<LOQ, that is, <10 IU/ml.

were compared using the X^2 test or Fisher's exact test, and continuous variables were compared using Student *t* test, the Mann–Whitney *U* test, or the Kruskal–Wallis test, as appropriate. All tests were two-sided and used a significance level of 0.05.

Results

Overall patient characteristics

A total of 431 frozen plasma samples, collected from 130 patients with CHD (69 untreated and 61 BLV-treated), were analyzed. The baseline demographic, virological, and clinical features of the 130 patients with CHD are presented in Table 1 and Table S1. Most patients were middle-aged Caucasian males with compensated cirrhosis receiving nucleos(t)ide analog (NUC) therapy for HBV. HDV genotyping was performed in 115 (88%) patients with HDV RNA levels high enough to perform this analysis: 111 (97%) patients were genotype 1 and four (3%) patients were genotype 5. Median alanine aminotransferase (ALT) levels were 76 (6–743) U/L, HBsAg was 3.8 (0.3–4.6) log IU/ml, 87% of patients were HBeAg-negative, and 73% had undetectable HBV DNA.

WHO HDV RNA International Standard

Tables 2 and 3 summarize the performances of the three assays compared with the WHO HDV RNA International Standard. Robogene 2.0 read a median of 0.20 log IU/ml below the expected values in five out of six samples; however, the last sample, which had an expected value of 7 IU/ml, was below the LOD with Robogene 2.0. EurobioPlex read a median of 0.66 log IU/ml more than the expected values in four out of six samples; however, two of these samples were <100 IU/ml with the WHO International Standard, resulting in below the LOD with EurobioPlex. Conversely, AltoStar read a median of 0.12 log IU/ml more than the WHO International Standard, with no difference for samples falling below the LOD.

Robogene 2.0 *vs.* EurobioPlex

HDV RNA levels in the overall cohort of patients with CHD (untreated and BLV-treated)

Overall, 232 samples were tested using Robogene 2.0 and EurobioPlex. Median HDV RNA levels measured by Robogene 2.0 *vs.* EurobioPlex were 3.78 (0.70–7.99) *vs.* 4.69 (2.00–8.19) IU/ml, respectively (p <0.0001) (Table 4 and Fig. 1A). This difference was confirmed when only genotype 1 samples were considered (Table 4). Compared with Robogene 2.0, EurobioPlex reported similar HDV RNA levels (Δ ± 0.5 log) in 66 (28%) patients, higher levels by >0.5 log IU in 160 (69%), and lower levels in only 6 (3%) (Fig. 1B). Of the 40 (17%) samples that tested target not detected (TND) by Robogene 2.0, 29 (73%) were also identified as TND by EurobioPlex, five (13%)

Table 2. WHO International Standards for HDV RNA quantification tested by Robogene 2.0 and EurobioPlex.

WHO HDV standard	HDV RNA levels WHO (log IU/ml)	Robogene 2.0 (log IU/ml)	ΔWHO *vs.* Robogene 2.0 (log IU/ml)	EurobioPlex (log IU/ml)	ΔWHO *vs.* EurobioPlex (log IU/ml)
S1	5.76	5.16	-0.60	6.42	0.66
S2	4.76	4.56	-0.20	5.44	0.68
S3	3.76	3.61	-0.15	4.37	0.61
S4	2.76	2.57	-0.19	3.26	0.50
S5	1.76	1.30	-0.46	2.00	–
S6	0.76	0.70	0	2.00	–

WHO, World Health Organization.

Table 3. WHO International Standards for HDV RNA quantification tested by AltoStar.

WHO HDV standard	HDV RNA levels WHO (log IU/ml)	AltoStar (log IU/ml)	ΔWHO *vs.* AltoStar (log IU/ml)
S1	5.70	–	–
S2	4.70	4.76	-0.06
S3	3.70	3.84	-0.14
S4	2.70	2.86	-0.16
S5	1.70	1.82	-0.12
S6	0.70	0.82	-0.12

WHO, World Health Organization.

Table 4. HDV RNA levels according to HDV assays and treatment status.

Samples group	n	Robogene 2.0 HDV RNA (log IU/ml)	EurobioPlex HDV RNA (log IU/ml)	*p* value	n	Robogene 2.0 HDV RNA (log IU/ml)	AltoStar HDV RNA (log IU/ml)	*p* value
Overall	232	3.78 (0.70–7.99)	4.69 (2.00–8.19)	<0.0001	246	3.32 (0.70–7.37)	3.91 (0.19–7.54)	<0.0001
Untreated patients*	109	4.75 (0.70–7.99)	5.59 (2.00–8.19)	<0.0001	49	5.11 (2.42–7.37)	5.63 (2.79–7.54)	<0.0001
BLV-treated patients	123	2.59 (0.70–6.44)	3.11 (2.00–8.04)	<0.0001	197	2.75 (0.70–6.80)	3.40 (0.19–7.41)	<0.0001
Genotype 1 samples	221	3.83 (0.70–7.99)	4.72 (2.00–8.19)	<0.0001	238	3.32 (0.70–7.37)	3.91 (0.19–7.54)	<0.0001

Results are reported as median (range). Continuous variables were compared using the paired-samples *t* test.

*Included baseline samples from BLV-treated patients for Robogene 2.0 *vs.* EurobioPlex and only baseline samples from BLV-treated patients for Robogene 2.0 *vs.* AltoStar. BLV, bulevirtide.

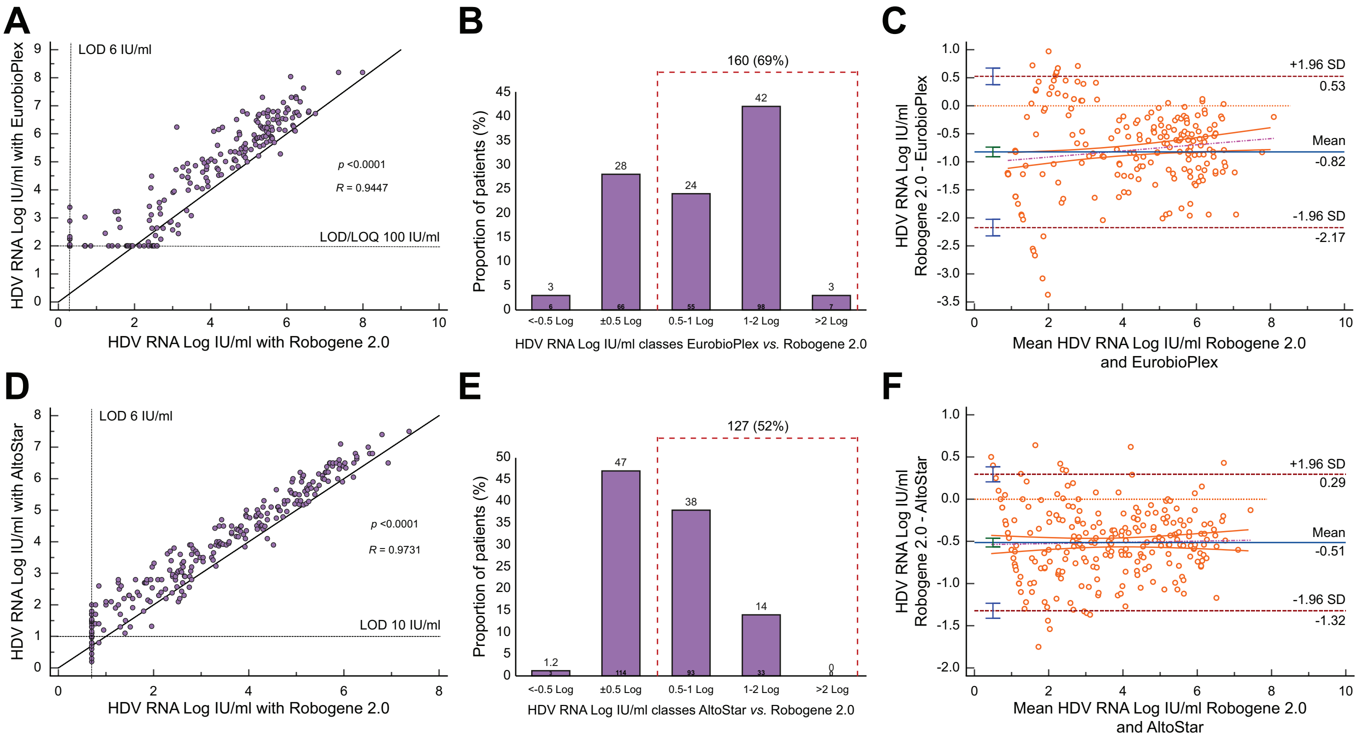

Fig. 1. Correlation between HDV RNA levels quantified by different assays. (A) Scatter diagram correlation between Robogene 2.0 and EurobioPlex. (B) Differences in HDV RNA levels between Robogene 2.0 and EurobioPlex. (C) Scatter diagram correlation between Robogene 2.0 and Altostar. (D) Differences in HDV RNA levels between Robogene 2.0 and AltoStar. (E) Bland–Altman plot comparing HDV RNA levels quantified by Robogene 2.0 and EurobioPlex. (F) Bland–Altman plot comparing HDV RNA levels quantified by Robogene 2.0 and AltoStar. LOD, lower limit of detection; LOQ, lower limit of quantification.

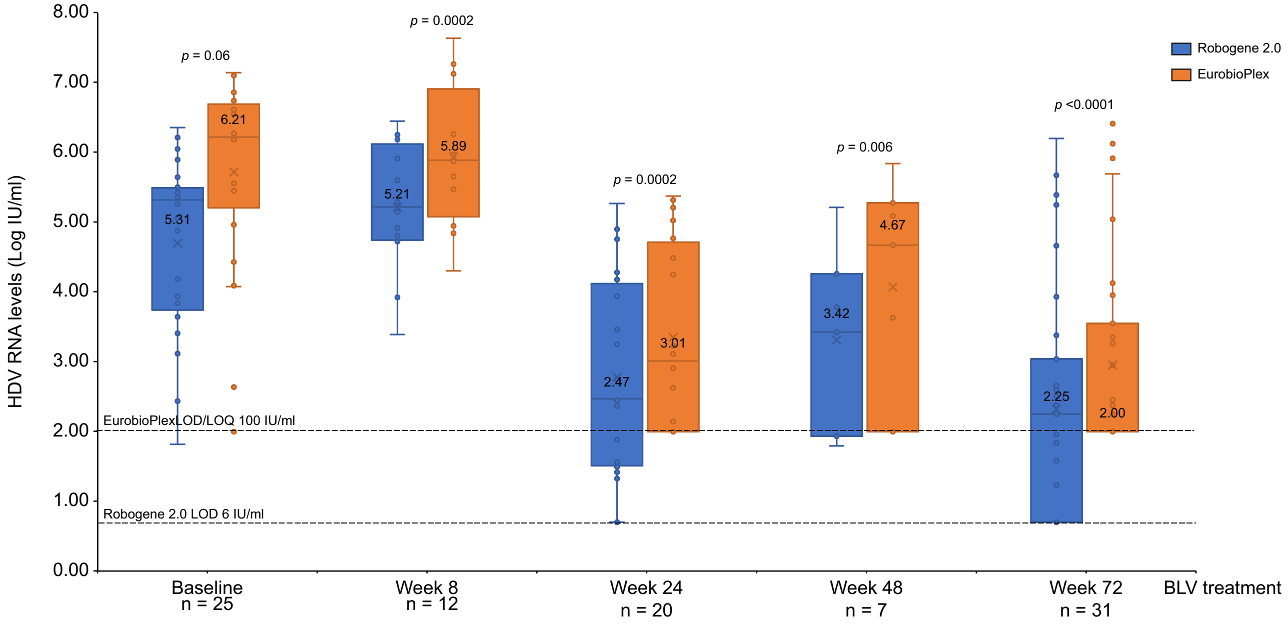

N° of patients with	Baseline	Week 8	Week 24	Week 48	Week 72
HDV RNA TND/<LOD	0 (0%) 1 (4%)	0 (0%) 0 (0%)	2 (10%) 7 (35%)	0 (0%) 2 (29%)	11 (36%) 16 (52%)
HDV RNA TND	0 (0%) 1 (4%)	0 (0%) 0 (0%)	1 (5%) 4 (25%)	0 (0%) 1 (14%)	4 (13%) 10 (32%)

Fig. 2. Box and whisker graph showing HDV RNA levels and HDV RNA undetectability rates tested using Robogene 2.0 and EurobioPlex in patients with CHD treated with BLV 2 mg monotherapy for 72 weeks. Comparison across assays of continuous variables using the paired-samples *t* test. BLV, bulevirtide; LOD, lower limit of detection; TND, target not detected.

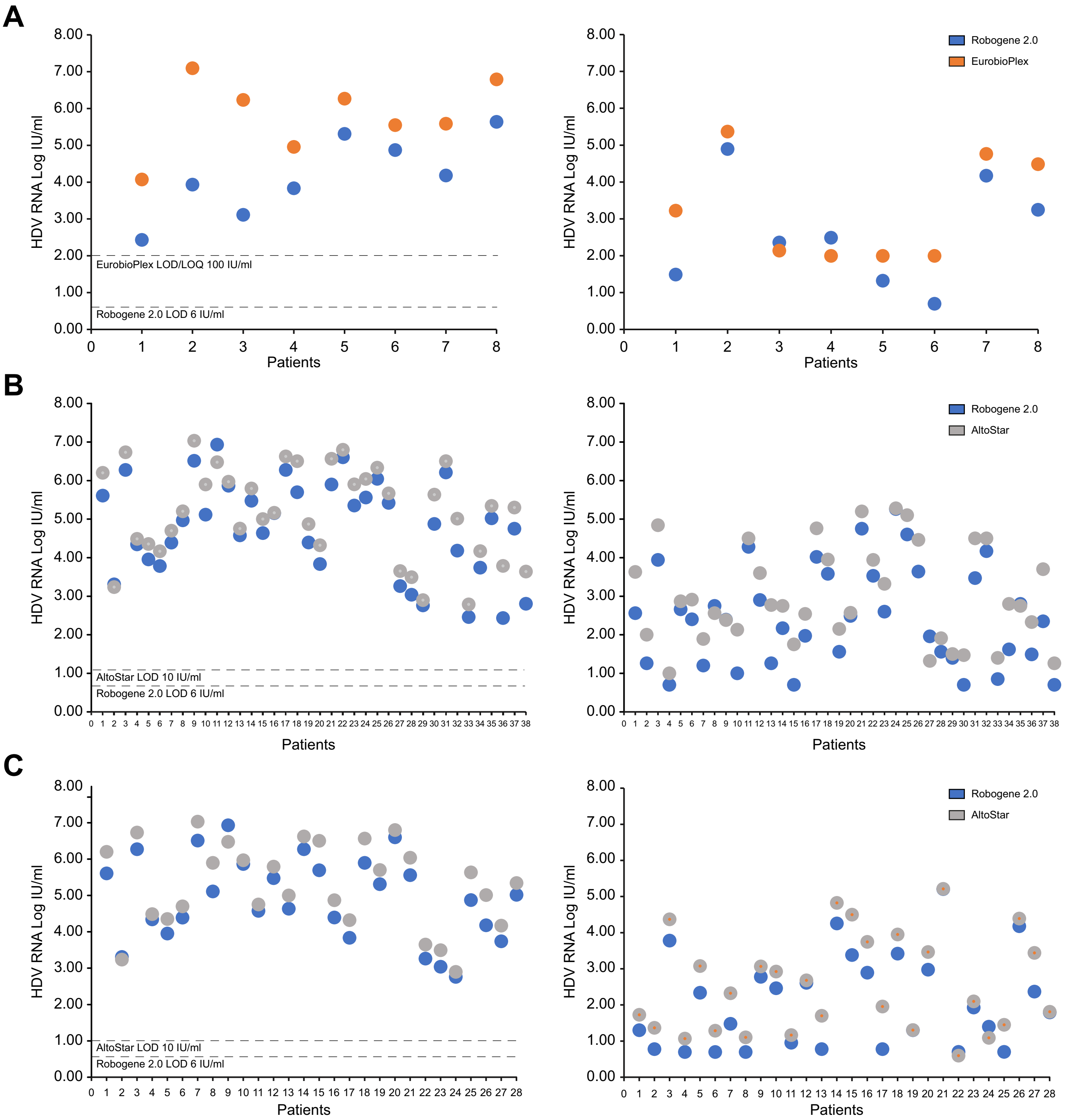

Fig. 3. HDV RNA levels quantified using Robogene 2.0 *vs.* EurobioPlex and using Robogene 2.0 *vs.* AltoStar in patients treated with BLV. Subanalysis in paired baseline and Week 24 samples and in paired baseline and Week 48 samples. (A) Baseline (left) and Week 24 (right) HDV RNA levels tested by Robogene 2.0 (blue) *vs.* EurobioPlex (orange). (B) Baseline (left) *vs.* Week 24 (right) HDV RNA levels tested by Robogene 2.0 (blue) *vs.* AltoStar (gray). (C) Baseline (left) *vs.* Week 48 (right) HDV RNA levels tested by Robogene 2.0 (blue) *vs.* AltoStar (gray). BLV, bulevirtide.

were below the LOD/LOQ, and six (15%) tested positive. Of the 14 (6%) samples reported below the LOD by Robogene 2.0, 11 (79%) were TND with EurobioPlex, two (14%) were below the LOD/LOQ, and one was detected positive (Table S2a).

Of 232 plasma samples, 109 (47%) were collected from untreated patients with CHD. Median HDV RNA levels were 4.75 (0.70–7.99) log IU/ml with Robogene 2.0 and 5.59 (2.00–8.19) with EurobioPlex (p <0.0001) (Table 4). The median difference between the two tests was 0.84 (-0.49 to 2.16) log IU/ml. Compared with the Robogene 2.0 test, EurobioPlex reported similar HDV RNA levels (Δ ± 0.5 log) in 26 (31%) patients, higher in 56 (67%), and lower in three (4%).

A total of 123 (53%) plasma samples were collected from BLV-treated patients. Median HDV RNA levels were 2.59 (0.70–6.44) log IU/ml and 3.11 (2.00–8.04) log IU/ml with Robogene 2.0 and EurobioPlex, respectively (p <0.0001) (Table 4). The difference in HDV RNA quantification between the two tests persisted at all time points—from baseline to Week 72—of BLV therapy (Fig. 2). We then evaluated the achievement of a virological response, defined as an HDV RNA decrease of ≥2 log from baseline or HDV RNA undetectability, in a subset of patients (n = 9) for whom paired baseline and Week 24 data were available. Three (33%) *vs.* five (55%) patients achieved a virological response with Robogene 2.0 *vs.* EurobioPlex, respectively (p = 0.31). Moreover, only one (11%) patient achieved HDV RNA undetectability by Week 24 with Robogene 2.0, whereas three (33%) patients achieved the same result with EurobioPlex at that time point (Fig. 3).

Robogene 2.0 *vs.* AltoStar

A total of 246 frozen plasma samples collected from 44 BLV-treated patients with CHD were analyzed. The baseline demographic, virological, and clinical features of these 44 patients with CHD are summarized in Table 1 and Table S1. Overall, median HDV RNA levels were 3.32 (0.70–7.37) *vs.* 3.91 (0.19–7.54) log IU/ml with Robogene 2.0 *vs.* AltoStar, respectively (p <0.0001) (Table 4). This difference was confirmed when only genotype 1 samples were considered (Table 4). The correlation between the two tests was excellent (R = 0.9731) (Fig. 1D). Compared with Robogene 2.0, AltoStar reported similar HDV RNA levels (Δ ± 0.5 log) in 114 (47%) patients, higher in 127 (52%), and lower in three (1.2%) (Fig. 1E). Of the nine (4%) samples that were identified as TND by Robogene 2.0, one (11%) tested TND also by AltoStar, four (44%) were below the LOD, and four (44%) tested positive. Of the 17 (7%) samples testing below the LOD by Robogene 2.0, none (0%) tested TND

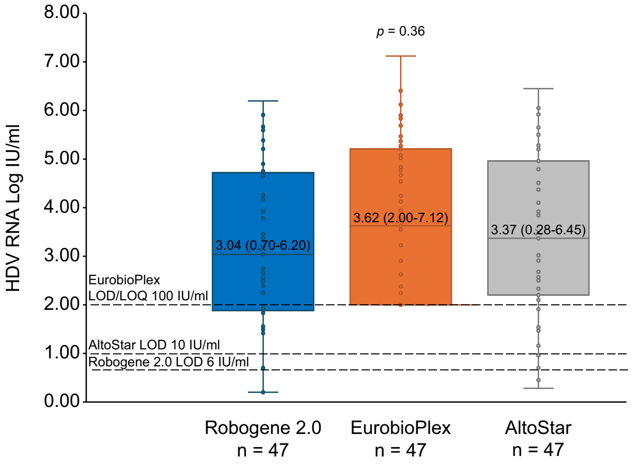

N° of patients with	Robogene 2.0	EurobioPlex	AltoStar
HDV RNA TND/<LOD	7 (15%)	16 (34%)	4 (9%)
HDV RNA TND	3 (6%)	8 (17%)	0 (0%)

Fig. 5. HDV RNA levels quantified using all three assays (Robogene 2.0, EurobioPlex, and AltoStar) in 47 samples. Comparison across assays of continuous variables using ANOVA. LOD, lower limit of detection; TND, target not detected.

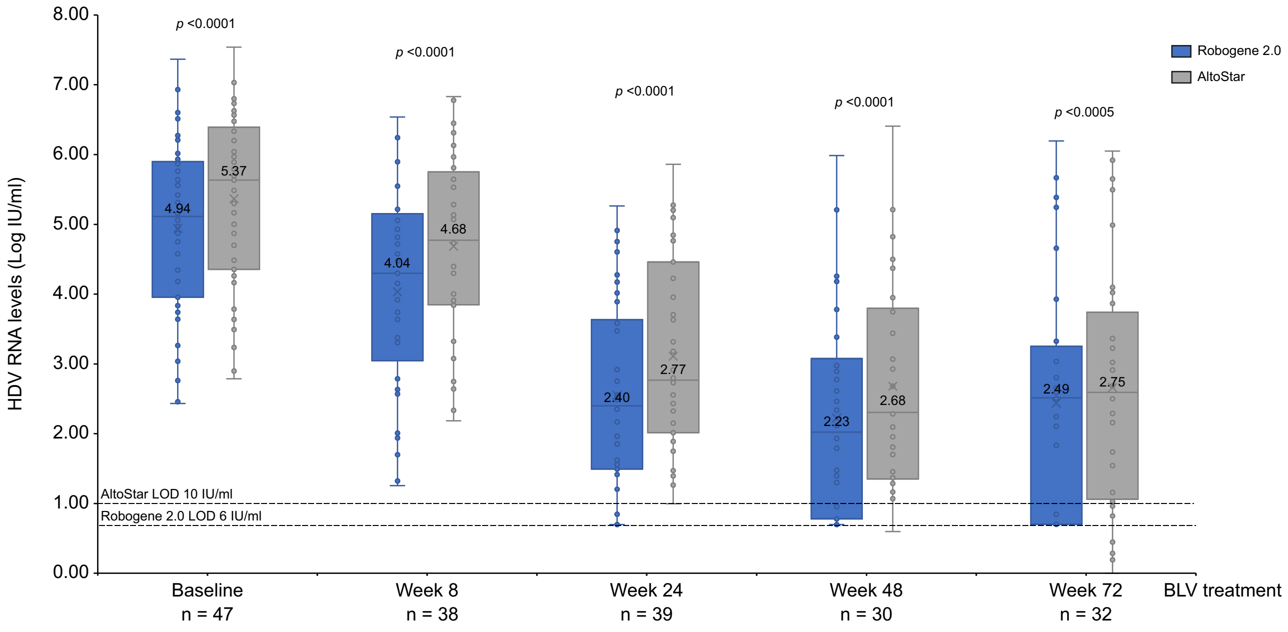

N° of patients with	Baseline	Week 8	Week 24	Week 48	Week 72
HDV RNA TND/<LOD	0 (0%) 0 (0%)	0 (0%) 0 (0%)	4 (10%) 1 (3%)	5 (17%) 1 (3%)	10 (31%) 7 (22%)
HDV RNA TND	0 (0%) 0 (0%)	0 (0%) 0 (0%)	1 (3%) 0 (0%)	2 (7%) 0 (0%)	5 (16%) 1 (3%)

Fig. 4. Box and whisker graph showing HDV RNA levels and HDV RNA undetectability rates assessed using Robogene 2.0 or AltoStar in patients with CHD treated with BLV 2 mg monotherapy for 72 weeks. Comparison across assays of continuous variables using the paired-samples t test. BLV, bulevirtide; LOD, lower limit of detection; TND, target not detected.

with AltoStar, three (18%) tested below the LOD, and 14 (82%) were positive (Table S2b).

Forty-nine samples were collected at BLV treatment baseline. Median HDV RNA levels were 5.11 (2.42–7.37) log IU/ml with Robogene 2.0 and 5.63 (2.79–7.54) log IU/ml with AltoStar (p <0.0001). The difference between the two tests remained statistically significant at all timepoints during BLV therapy (p <0.0001 or p = 0.0005). The widest difference between tests was observed at Week 8, whereas the narrowest was observed at Week 72 (Fig. 4).

We then evaluated the achievement of virological response, defined as an HDV RNA decrease of ≥2 log from baseline or HDV RNA undetectability, in a subset of patients (n = 38) for whom paired baseline and Week 24 data were available. In total, 17 patients achieved a virological response with both Robogene 2.0 and AltoStar at Week 24. One patient (patient 27) achieved a virological response with AltoStar but not with Robogene 2.0. Eight patients achieved a virological response with Robogene 2.0 but not with AltoStar. Overall, virological response rates did not differ significantly between the two assays (p = 0.13) (Fig. 3B). As of Week 48, 28 paired samples were available. In addition, 19 patients achieved a virological response with both Robogene 2.0 and AltoStar, no patient achieved a virological response with AltoStar but not with Robogene 2.0, and two patients achieved a virological response with Robogene 2.0 but not with AltoStar. Again, virological response rates did not differ significantly between the two tests (p = 0.77) (Fig. 3C).

Robogene 2.0 *vs.* EurobioPlex *vs.* AltoStar

In 47 samples collected from BLV-treated patients, HDV RNA levels were quantified using all three assays. Median HDV RNA values were 3.04 (0.70–6.20) *vs.* 3.62 (2.00–7.12) *vs.* 3.37 (0.28–6.45) IU/ml with Robogene 2.0 *vs.* EurobioPlex *vs.* AltoStar, respectively (p = 0.36) (Fig. 5). A total of 16 samples tested negative with EurobioPlex; of these, 10 (63%) were positive with Robogene 2.0 and three (19%) with AltoStar. Seven samples tested negative with Robogene 2.0; of these, all but one were also negative with EurobioPlex, whereas three were positive with AltoStar. Fifteen samples were from Week 24: HDV RNA was undetectable in 0, five (33%), and 0 patients with Robogene 2.0, EurobioPlex, and AltoStar, respectively (p = 0.003). Furthermore, 14 samples were from Week 72: HDV RNA was undetectable in five (36%), 10 (71%), and three (21%) of patients with Robogene 2.0, EurobioPlex, and AltoStar, respectively (p = 0.02).

Discussion

In this single-center retrospective study, we demonstrated that quantification of HDV RNA levels in untreated and BLV-treated patients with CHD is significantly influenced by the quantification method used. Compared with Robogene 2.0, EurobioPlex significantly overestimated viremia by approximately 1 log. A similar observation was made by comparing EurobioPlex with the WHO International Standard for HDV RNA. Quantification of HDV RNA levels by AltoStar significantly correlated with those measured by Robogene 2.0, but viremia was half a log IU/ml higher with the former assay. Although virological response rates during BLV monotherapy did not differ between the three assays, HDV RNA undetectability rates did.

Viremia monitoring holds a position of utmost significance in both the diagnosis and management of untreated and treated individuals with viral hepatitis. The topic gains even more importance in the context of CHD, where the absence of a universally accepted assay for viral load quantification poses a substantial challenge.[22] Different centers rely upon different commercial or in-house assays, amplifying the complexity of results interpretation. The consequences of this variability become particularly pronounced when addressing HDV genotypes other than genotype 1, which is the only one with a WHO International Standard for HDV RNA.

Up until now, no study has compared the performances of Robogene 2.0, EurobioPlex, and AltoStar, which are among the most popular available assays in Europe to quantify HDV viremia. Two studies suggested that HDV RNA quantification might be significantly influenced by the extraction methods, even with the same quantification assay, the latter of the two evaluating HDV RNA in both untreated and BLV-treated patients.[14,21] It is worth highlighting that both studies used Robogene 2.0, which is the assay used in the phase III registration MYR301 study for BLV.[20] Therefore, this observation could lead to important consequences, as virological response to BLV is one of the endpoints that led to its EMA approval.[11] In contrast, EurobioPlex has been widely used in other studies involving patients with CHD treated with BLV.[23,24] A recently developed version of the EurobioPlex quantification assay shows high reproducibility and repeatability when compared with the previous version, although it reads 0.88 log IU/ml lower than the latter. This new version aligns closely with theoretical values, according to WHO International Standards.[19]

Our study provided new insights into the differences in HDV RNA quantification by different assays. We included a large cohort of patients with CHD, with a significant proportion of patients being treated with BLV at the time of the study, and demonstrated that EurobioPlex generally measures higher values of HDV RNA compared with Robogene 2.0. However, when Robogene 2.0 is compared to AltoStar, the two tests correlate very well and the difference between the two tests is approximately 0.5 log IU/ml. This observation held true also when quantification with the three tests was performed with the WHO HDV RNA International Standard. In contrast to what Gerber *et al.*[19] observed for the new version of the EurobioPlex assay, in our study, EurobioPlex read 0.5 log IU/ml more than the WHO International Standard theoretical value.

To our knowledge, this is the first study evaluating discrepancies in HDV RNA quantification with three European conformity (CE)-labeled or RUO HDV RNA assays in patients with CHD treated with BLV. The differences in HDV RNA levels quantification between the three tests persisted in patients treated with BLV for up to 72 weeks. Although virological response rates at Week 24 (for Robogene 2.0 *vs.* EurobioPlex and for Robogene 2.0 *vs.* AltoStar) and at Week 48 (for Robogene 2.0 *vs.* AltoStar) of BLV therapy were similar between the assays, the rates of HDV RNA undetectability significantly differed. The proportion of patients achieving negative or very low viremia by EurobioPlex was approximately three times greater than that observed by the other two tests. As EurobioPlex is a very popular assay in France, these findings may explain, at least in part, why the rates of negative viremia in all the real-life BLV studies in France are higher than those observed by the same treatment in similar patients in countries

where different assays are used.[24] The different sensitivity of these assays may have important implications also for the management of BLV monotherapy, the only approved therapy in the EU for compensated CHD. Because discontinuation of BLV monotherapy can be considered only in patients who achieve and maintain long-term undetectable viremia, the use of low-sensitivity HDV RNA quantification assays may lead to BLV discontinuation in patients who are still HDV RNA positive at low levels. This could lead to important consequences, such as a high risk of viral reactivation upon therapy withdrawal.[25–27]

Some of the strengths of our study are the size of the cohort enrolled, the use of two commercially available CE-labeled assays and one RUO assay rather than in-house methods, and the selection of patients who were untreated and treated with BLV. As this was a monocentric study, plasma samples were all processed in the same laboratory following the same protocol, thus reducing variability. One of the major limitations of our study is the type of cohort enrolled: the vast majority of our patients were infected with HDV genotype 1, so we cannot anticipate if our observations could be generalized to other clinical settings. The predominance of genotype 1 samples is in line with a recent multicenter epidemiological nationwide Italian study, which observed that most of untreated patients with CHD belonged to genotype 1.[28] Likewise, more than 90% of the patients enrolled in the BLV registration studies were infected with genotype 1.[20,29] Because the number of non-genotype 1 samples is limited, no conclusions can be safely drawn regarding the performances of the three HDV RNA assays in these samples.

Moreover, the number of patients for whom paired baseline and Week 24 and 48 data were available is limited, so the lack of statistical significance could be attributed to sample size. Further studies are needed to assess the real impact of the assay on defining virological response to BLV therapy.

In conclusion, this is the first study to highlight the differences in HDV RNA quantification by three CE-labeled quantification methods and one RUO HDV RNA quantification method in a large cohort of patients with CHD. HDV RNA levels significantly differed across different assays in untreated and BLV-treated patients with CHD, highlighting the need for new studies aimed at further improving the quantification of HDV RNA levels.

Affiliations

[1]*Division of Gastroenterology and Hepatology, Foundation IRCCS Ca' Granda Ospedale Maggiore Policlinico, Milan, Italy;* [2]*D-SOLVE Consortium, an EU Horizon Europe Funded Project (No. 101057917), Hannover, Germany;* [3]*Microbiology and Virology Unit, Foundation IRCCS Ca' Granda Ospedale Maggiore Policlinico, Milan, Italy;* [4]*Department of Pathophysiology and Transplantation, CRC "A. M. and A. Migliavacca" Center for Liver Disease, University of Milan, Milan, Italy*

Abbreviations

ALT, alanine aminotransferase; anti-HD, hepatitis delta antigen antibody; AST, aspartate aminotransferase; BLV, bulevirtide; CHD, chronic hepatitis delta; LOD, lower limit of detection; LOQ, lower limit of quantification; LSM, liver stiffness measurement; NUC, nucleos(t)ide analog; PegIFNα, pegylated interferon alpha; PLT, platelets; qHBsAg, quantitative HBsAg; RUO, research use only; WHO, World Health Organization.

Financial support

The present research and preparation of the article did not receive any financial support.

Conflicts of interest

ED is an advisory board member for AbbVie and receives speaking and teaching fees from Gilead, MSD, and AbbVie. PL is an advisor and speaker bureau for Roche Pharma/Diagnostics, Gilead Sciences, Gsk, AbbVie, Janssen, Myr, Eiger, Antios, Aligos, Vir, Grifols, Altona, and Roboscreen. The other authors have nothing to disclose.

Please refer to the accompanying ICMJE disclosure forms for further details.

Authors' contributions

Concept and design: ED, SUR, MPA, PL. Data collection: ED, MPA, SM, MB, RP, DS, FF. Writing of the article: MPA, ED, PL. Statistical analysis: MPA, ED, PL. Virological analysis: SUR, AC. Critical revision of the manuscript: AC, PL. Approved the final version of the manuscript: all authors.

Acknowledgements

The authors acknowledge support from the University of Milan.

Supplementary data

Supplementary data to this article can be found online at https://doi.org/10.1016/j.jhepr.2024.101299.

References

Author names in bold designate shared co-first authorship

[1] Stockdale AJ, Kreuels B, Henrion MYR, et al. The global prevalence of hepatitis D virus infection: systematic review and meta-analysis. J Hepatol 2020;73:523–532.

[2] Miao Z, Zhang S, Ou X, et al. Estimating the global prevalence, disease progression, and clinical outcome of Hepatitis Delta Virus infection. J Infect Dis 2020;221:1677–1687.

[3] Fattovich G, Boscaro S, Noventa F, et al. Influence of hepatitis delta virus infection on progression to cirrhosis in chronic hepatitis type B. J Infect Dis 1987;155:931–935.

[4] Rosina F, Conoscitore P, Cuppone R, et al. Changing pattern of chronic hepatitis D in Southern Europe. Gastroenterology 1999;117:161–166.

[5] Wedemeyer H, Yurdaydin C, Hardtke S, et al. Peginterferon alfa-2a plus tenofovir disoproxil fumarate for hepatitis D (HIDIT-II): a randomized, placebo controlled, phase 2 trial. Lancet Infect Dis 2019;19:275–286.

[6] European Association for the Study of the Liver. EASL clinical practice guidelines on the management of hepatitis B virus infection. J Hepatol 2017;67:370–398.

[7] European Association for the Study of the Liver. EASL recommendations on treatment of hepatitis C. https://www.easl.eu/medias/cpg/HCV2016/English-report.pdf. [Accessed 22 January 2025].

[8] Romeo R, Foglieni B, Casazza G, et al. High serum levels of HDV RNA are predictors of cirrhosis and liver cancer in patients with Chronic Hepatitis Delta. PLoS One 2014;9:e92062.

[9] Keskin O, Wedemeyer H, Tüzün A, et al. Association between level of hepatitis D virus RNA at week 24 of pegylated interferon therapy and outcome. Clin Gastroenterol Hepatol 2015;13:2342–2349.e1-2.

[10] Kamal H, Westman G, Falconer K, et al. Long-term study of hepatitis delta virus infection at secondary care centers: the impact of viremia on liver-related outcomes. Hepatology 2020;72:1177–1190.

[11] Cornberg M, Lok AS, Terrault NA, et al. 2019 EASL-AASLD HBV Treatment Endpoints Conference Faculty. Guidance for design and endpoints of clinical trials in chronic hepatitis B—report from the 2019 EASL-AASLD HBV Treatment Endpoints Conference. J Hepatol 2020;72:539–557.

[12] Yurdaydin C, Abbas Z, Buti M, et al. Treating chronic hepatitis delta: the need for surrogate markers of treatment efficacy. J Hepatol 2019;70:1008–1015.

[13] Stelzl E, Ciesek S, Cornberg M, et al. Reliable quantification of plasma HDV RNA is of paramount importance for treatment monitoring: a European multicenter study. J Clin Virol 2021;142:104932.

[14] Bremer B, Anastasiou OE, Ciesek S, et al. Automated nucleic acid isolation methods for HDV viral load quantification can lead to viral load underestimation. Antivir Ther 2019;24(2):117–123.

[15] Wedemeyer H, Leus M, Battersby TR, et al. HDV RNA assays: performance characteristic, clinical utility, and challenges. Hepatology 2025;81(2):637–650.

[16] Chudy M, Hanschmann KM, Bozdayi M, et al. Collaborative study to establish a World Health Organization international standard for hepatitis D

virus RNA for nucleic acid amplification technique (NAT)-based assays. World Health Organization; 2013. https://apps.who.int/iris/bitstream/handle/10665/96341/WHO_BS_2013.2227_eng.pdf?sequence=1&isAllowed=y. [Accessed 22 January 2025].

[17] Pflüger LS, Nörz D, Volz T, et al. Clinical establishment of a laboratory developed quantitative HDV PCR assay on the cobas6800 high-throughput system. JHEP Rep 2021;3:100356.

[18] **Urban S**, **Neumann-Haefelin C**, Lampertico P. Hepatitis D virus in 2021: virology, immunology and new treatment approaches for a difficult-to-treat disease. Gut 2021;70:1782–1794.

[19] Gerber A, Huault E, Delagarde V, et al. Evaluation of EurobioPlex EBX-071, a new commercial kit for HDV viral load quantification. Hepatology 2023;78:S380–S381.

[20] Wedemeyer H, Aleman S, Brunetto MR, et al. A phase 3, randomized trial of bulevirtide in chronic hepatitis D. N Engl J Med 2023;389:22–32.

[21] Anolli MP, Uceda Renteria S, Degasperi E, et al. Quantification of serum HDV RNA by Robogene 2.0 in HDV patients is significantly influenced by the extraction methods. Liver Int 2024;44:831–837.

[22] Le Gal F, Brichler S, Sahli R, et al. First international external quality assessment for hepatitis delta virus RNA quantification in plasma. Hepatology 2016;64:1483–1494.

[23] De Lédinghen V, Bardou-Jacquet E, Metivier S, et al. HDV-RNA decline less than 1 log after 6 months of BLV 2 mg monotherapy could define poor-response and lead to therapeutic decision. Data from real-life cohort. J Hepatol 2023;78:S56–S57.

[24] De Lédinghen V, Hermabessière P, Metivier S, et al. Bulevirtide, with or without PEG-Interferon, in HDV infected patients in a real-life setting. Two years results from the French multicenter early access program. Hepatology 2022;76:S26–S27.

[25] Anolli MP, Degasperi E, Allweiss L, et al. A 3-year course of bulevirtide monotherapy may cure Hdv infection in cirrhotics. J Hepatol 2023. S0168-8278(22)03475-4. Epub ahead of print.

[26] Jachs M, Panzer M, Hartl L, et al. Long-term follow-up of patients discontinuing bulevirtide treatment upon long-term HDV-RNA suppression. JHEP Rep 2023;5:100751.

[27] Heidrich B, Yurdaydın C, Kabaçam G, et al. Late HDV RNA relapse after peginterferon alpha-based therapy of chronic hepatitis delta. Hepatology 2014;60:87–97.

[28] Caviglia GP, Colombatto P, Coco B, et al. Burden of hepatitis D virus infection in Italy: interim results from a prospective multicentre nationwide study. Dig Liv Dis 2024;56:S16.

[29] Asselah T, Chulanov V, Lampertico P, et al. Bulevirtide combined with pegylated interferon for chronic hepatitis D. N Engl J Med 2024;391:133–143.

Keywords: HDV; HDV RNA; PCR; Chronic hepatitis delta; Nucleic acid; Bulevirtide; EurobioPlex; AltoStar; Robogene; Antiviral therapy.

Received 23 April 2024; received in revised form 3 December 2024; accepted 5 December 2024; Available online 11 December 2024

Journal of Hepatology, Volume *7*

Supplemental information

Comparing methods for plasma HDV RNA quantification in bulevirtide-treated and untreated patients with HDV

Maria Paola Anolli, Sara Uceda Renteria, Elisabetta Degasperi, Floriana Facchetti, Dana Sambarino, Marta Borghi, Riccardo Perbellini, Roberta Soffredini, Sara Monico, Annapaola Callegaro, and Pietro Lampertico

Comparing methods for plasma HDV RNA quantification in bulevirtide-treated and untreated patients with HDV

Maria Paola Anolli, Sara Uceda Renteria, Elisabetta Degasperi, Floriana Facchetti, Dana Sambarino, Marta Borghi, Riccardo Perbellini, Roberta Soffredini, Sara Monico, Annapaola Callegaro, Pietro Lampertico

Table S1……………………………………………………………………………..…..2

Table S2a……………………………………………………………………………..4

Table S2b……………………………………………………………………………..4

Table S1. Main demographic, biochemical and virological features of the 130 patients included in the study

**Variables**	**Robogene *vs.* EurobioPlex Patients n=127**	**Robogene *vs.* AltoStar Patients n=44**
Age, years	52 (23-77)	51 (29-77)
Males	68 (54%)	25 (57%)
BMI, kg/m^2	24 (17-41)	24 (19-41)
European origin	116 (91%)	42 (96%)
HDV Genotype 1*	108 (96%)*	44 (100%)*
Compensated cirrhosis†	79 (62%)	44 (100%)
Ongoing NUC treatment	95 (75%)	44 (100%)
Previous PegIFNα treatment▪	63 (50%)	23 (52%)
AST, U/L	63 (16-374)	46 (18-592)
ALT, U/L	76 (6-743)	43 (12-684)
PLT, $10^3/mm^3$	146 (29-344)	80 (37-220)
LSM kPa°	11.1 (4.0-62.1)	16.5 (7.2-62.1)
Esophageal varices§	30 (41%)§	24 (57%)
qHBsAg, Log IU/mL	3.8 (0.3-4.6)	3.7 (0.3-4.4)
HbeAg negative	111 (87%)	42 (95%)
HBV DNA undetectable✡	93 (73%)	42 (95%)

*Genotype available in 112 (89%) and 44 (100%) patients

† Child Pugh Score > A in 11 (14%) patients

° Fibroscan® available in 102 (80%) and 44 (100%) patients

§EGD available in 73 (58%) and 44 (100%) patients

✡ <LLOQ, i.e. <10 UI/mL

Results are reported as number (percentage) or median (range).

BMI, body mass index; HDV, Hepatitis D Virus; NUC, nucleos(t)ide analogue; PegIFNα, Pegylated Interferon alpha; AST, aspartate aminotransferase; ALT, alanine aminotransferase; PLT, platelets; LSM, liver stiffness measurement; qHBsAg, Hepatitis B surface Antigen; HbeAg, Hepatitis B e-Antigen

Table S2a. HDV RNA levels quantified by both Robogene 2.0 and EurobioPlex in 232 samples

	**EurobioPlex**		
**Robogene 2.0**	**TND (n=39)**	**<LOD/LOQ (n=20)**	**DETECTED (n=166)**
TND (n=40)	29 (73%)	5 (13%)	6 (15%)[1]
<LOD (n=14)	11 (79%)	2 (14%)	1 (7%)[2]
Detected (n=178)	6 (3%)[3]	13 (7%)[4]	159 (89%)

[1] Median HDV RNA 2.29 (2.02-3.38) Log IU/mL; [2] HDV RNA 3.30 Log IU/mL; [3] Median HDV RNA 1.80 (1.32-2.49) Log IU/mL; [4] Median HDV RNA 2.25 (0.85-2.59) Log IU/ml

Table S2b. HDV RNA levels quantified by both Robogene 2.0 and AltoStar in 246 samples

	**AltoStar**		
**Robogene 2.0**	**TND (n=1)**	**<LOD (n=8)**	**DETECTED (n=237)**
TND (n=9)	1 (11%)	4 (44%)	4 (44%)[1]
<LOD (n=17)	0 (0%)	3 (18%)	14 (82%)[2]
Detected (n=220)	0 (0%)	1 (0.5%)[3]	219 (99.5%)

[1]Median HDV RNA 1.50, [2]median HDV RNA levels of 1.34 (1.00-2.03) Log IU/mL, [3]HDV RNA 0.85 IU/mL
